# Therapeutic Effects and Repair Mechanism of HGF Gene-Transfected Mesenchymal Stem Cells on Injured Endometrium

**DOI:** 10.1155/2022/5744538

**Published:** 2022-04-05

**Authors:** Xuan Xu, Qiong Xing, Ruijun Liu, Liu Dong, Zhen Yu, Ying Wang, Ping Zhou, Ying V. Zhang, Jianye Wang, Yunxia Cao, Zhaolian Wei

**Affiliations:** ^1^Reproductive Medicine Center, Department of Obstetrics and Gynecology, First Affiliated Hospital of Anhui Medical University, No 218 Jixi Road, Hefei, 230022 Anhui, China; ^2^NHC Key Laboratory of Study on Abnormal Gametes and Reproductive Tract, Anhui Medical University, No 81 Meishan Road, Hefei, 230032 Anhui, China; ^3^Key Laboratory of Population Health Across Life Cycle, Anhui Medical University, Ministry of Education of the People's Republic of China, No 81 Meishan Road, Hefei, 230032 Anhui, China; ^4^Anhui Province Key Laboratory of Reproductive Health and Genetics, No 81 Meishan Road, Hefei, 230032 Anhui, China; ^5^Nuwacell Biotechnologies Co., Ltd., No 2800 Chuangxin Avenue, Hefei, 230032 Anhui, China

## Abstract

There are many studies on the advantages of using mesenchymal stem cells (MSCs) that secrete various paracrine factors for repairing endometrial injury. However, the stability and effectiveness of MSCs require improvement to become a viable therapy. Hepatocyte growth factor (HGF), one of the cytokines secreted by MSCs, promotes vascular repair and mesenchymal to epithelial transformation (MET). Therefore, HGF likely promotes the repair process of the endometrium. We prepared MSCs transfected with the HGF gene to explore its repair effects and mechanism using a damaged endometrium mouse model. HGF gene-transfected MSCs were prepared by electroporation. The transfected MSCs retained their cellular characteristics and significantly increased the expression of HGF (*P* < 0.01). HGF gene-transfected MSCs helped damaged endometrium to recover its morphological characteristics, improved proliferation and decreased apoptosis of endometrial cells, increased the expression of endometrial vascular growth-related factors, and activated phosphorylated c-Met and AKT in the mouse endometrial damage model (*P* < 0.05). Compared with normal MSCs, HGF gene-transfected MSCs produced a more significant effect on damaged endometrial epithelium repair by activating the HGF/c-Met and downstream signaling pathways. Our results indicate that HGF gene-transfected MSCs provide an effective and promising tool for injured endometrium therapy.

## 1. Background

Endometrial injury, recognized as an important factor in implantation failure, commonly causes intrauterine adhesions (IUA) or endometrial thinning, leading to endometrial fibrosis, amenorrhea, infertility, or spontaneous abortion [1–3]. Conventional therapeutic methods, intrauterine implant positioning and high-dose hormone therapy, have been used to improve regeneration of the endometrium. However, the adverse effects, inefficiency, and postoperative recurrence of conventional therapy hinder the clinical application. Therefore, there is an urgent need to explore novel methods to repair endometrial damage, improve the endometrial microenvironment, and enhance the pregnancy rate [4–7].

Previous studies demonstrated that administration of mesenchymal stem cells (MSCs) promotes endometrial regeneration both morphologically and functionally, and also explored the underlying mechanism. Accumulating evidence shows that the functional benefit of transplanted MSCs is probably due to the paracrine effect of cytokines produced by transplanted MSCs [8–10]. To maintain the paracrine effect of MSCs, researchers have combined materials or pretreated MSCs with chemical drugs to promote endometrial regeneration after damage [11–13]. However, it may instead be more feasible to enhance the expression of reparative genes in transplanted MSCs and therefore synergistically restore endometrial function after injury.

Hepatocyte growth factor (HGF), a pleiotropic growth factor, is one of the many cytokines secreted by MSCs [14, 15]. HGF has a trophic effect on many types of cells, including epithelial, endothelial, and stromal cells, by promoting mitotic activity, growth, and migration and by preventing apoptosis [16]. A combination of cell and gene therapy strategies using MSCs genetically modified to overexpress HGF has been explored and achieved a better therapeutic effect than normal MSCs [17–19]. MSCs transfected with HGF have an antifibrotic effect in these diseases, which plays an important role in vascular repair and the mesenchymal epithelial transformation (MET) process. Similarly, the key mechanisms of endometrial repair are angiogenesis and the MET process. Therefore, MSCs transfected with HGF will likely enhance endometrial repair. In the current study, we transfected MSCs with the HGF gene to investigate the beneficial effects in an endometrial-injured mouse model and explored the mechanism of the endometrial reconstruction and regeneration.

## 2. Materials and Methods

### 2.1. Preparation of HGF Gene-Transfected MSCs

Human umbilical cords were obtained from women with full-term pregnancies at the First Affiliated Hospital of Anhui Medical University (Hefei, China). The ethical approval number for the human sample collection is PJ2018-01-07. The puerperae consented to donate and provided informed consent. The MSCs were isolated from whole umbilical cord using an explant culture method as described in a previous study [4]. Briefly, umbilical cord tissue was dissected, and the umbilical arteries, vein, and amniotic epithelium were gently removed, followed by three washes with sterile phosphate-buffered saline (PBS). The tissue was cultured with expansion medium consisting of Dulbecco's modified Eagle's medium (DMEM)/F-12 supplemented with 10% (*v*/*v*) fetal bovine serum (FBS) (Gibco, USA). After approximately 7–10 d, the adherent cells were harvested for subculturing.

MSCs at passage 3 were transfected with HGF minicircle deoxyribonucleic acid (DNA) vectors (mcHGF) using an electroporation system (Neon Transfection System) according to the manufacturer's instructions. The human mcHGF were produced as described by Kay et al. [20] and were generously provided by Nuwacell Biotechnologies Co., Ltd. MSCs were transfected with mcHGF by electroporation at 2 *μ* plasmid per 1.0 × 10^6^ cells (500 V, 2 pulses, 100 *μ*s). An mcHGF-green fluorescent protein (GFP) plasmid provided by Nuwacell Biotechnologies Co., Ltd., was also transfected under the same parameters and conditions, and the efficiency of transfection was assessed by fluorescence microscopy and flow cytometry at 12 h posttransfection.

### 2.2. Flow Cytometry (FCM)

Flow cytometry (FCM) was used to detect whether MSCs retained characteristic features following HGF gene transfection. The typical positive markers of MSCs were detected by a flow cytometer (BD FACS Calibur, USA) using antibodies for CD73 (1 : 1000, Abcam, UK), CD90 (1 : 1000, Abcam, UK), and CD105 (1 : 1000, Abcam, UK), conjugated with FITC or APC.

### 2.3. Enzyme-Linked Immunosorbent Assay (ELISA)

To analyze the amount of HGF expressed by transfected cells, culture media from MSCs transfected with minicircle vectors were analyzed by an enzyme-linked immunosorbent assay (ELISA) at 24, 48, 72, and 96 h posttransfection. The levels of HGF were quantified using a human HGFR Platinum ELISA (Affymetrix, San Diego, CA, USA), according to the manufacturer's instructions. After applying STOP solution to terminate the reaction, absorbance was measured at 450 nm.

### 2.4. Establishment of the Mouse Endometrial Injury Model

Eight-week-old female C57BL/6 mice (SPF level, Vital River Laboratory Animal Technology Co., Ltd.) were used in the study. They were housed five to six mice per cage at room temperature, with humidity 40% to 60%, light/dark cycle 12 h/12 h, and free access to food and water. The mice were anesthetized, and the uterine horns were exteriorized through a midline incision under sterile surgical conditions. Approximately 50 *μ*l of 95% ethanol was injected into the uterine horns until one side was filled, followed by three saline rinses. The uterus was gently repositioned and the wound was sutured. The mice then recovered from the anesthesia. Ethanol-treated experimental and saline-treated control mice were examined after 5 d to validate the establishment of the endometrial injury model.

### 2.5. Groups and Treatment

The groups are as follows: control group (did not undergo any treatment), saline group (saline-injected postinjury group), MSC group (MSC-injected postinjury group), and HGF gene-transfected MSCs (MSCs*^hgf^*) group (HGF gene-transfected MSC-injected postinjury group). The cells (2 × 10^5^) were injected in a total volume of 200 *μ*l into the tail vein on the fifth day after injury of the endometrium. Considering the half-life of cells *in vivo*, we euthanized the mice after 7 d and removed the uterine tissue. All experiments and methods were performed in accordance with the relevant ethical guidelines and regulations and were approved by the Experimental Center of the First Affiliated Hospital of Anhui Medical University. The approval number of the animal experiments is LLSC20190442.

### 2.6. Hematoxylin and Eosin (H&E) Staining

Uterine tissues were embedded in paraffin after fixing in 10% formalin. Sections were cut at 4 *μ*m thicknesses and stained with hematoxylin and eosin (H&E). The thickness of the endometrial epithelium and the number of glands were measured using an inverted phase contrast microscope.

### 2.7. Immunofluorescence Analysis

Sections of mouse uterus were fixed in precooled acetone and incubated with 0.3% hydrogen peroxide in 10% methanol for 15 min. After washing with PBS, the sections were blocked using an avidin/biotin blocking kit (Vector Laboratories, CA, USA). After incubation, blocking was performed using the Mouse on Mouse Fluorescein Kit (Vector Laboratories), following the manufacturer's instructions. Sections were incubated with anti-Ki-67 (1 : 300, Servicebio), anti-terminal deoxynucleotidyl transferase dUTP nick end labeling (TUNEL) (1 : 300, Elabscience), anti-E-cadherin (1 : 300, CST), and anti-vimentin (1 : 300, CST) overnight. In the next day, sections were incubated with rabbit anti-mouse IgG for 1 h. After DAPI staining, sections were observed with a confocal microscope (Carl Zeiss LSM800, Prenzlauer, Berlin, Germany). The average fluorescence intensity of immunofluorescence was measured using image analysis software (ZEN, Zeiss, Germany) and quantitatively analyzed.

### 2.8. Quantitative Reverse-Transcription Polymerase Chain Reaction (qRT-PCR)

Total RNA from mouse uteruses was extracted using TRIzol (Invitrogen, USA) according to the manufacturer's protocols. Next, 1 *μ*g of total RNA underwent reverse transcription to generate total cDNA using a reverse transcription kit (Transgen Biotech, Beijing, China) and oligo dT as the primer. Quantitative PCR was then performed using primers shown in Table 1 and FastStart Universal SYBR Green Master (Toyobo, Osaka, Japan), using a 7500 Real-Time PCR System (Applied Biosystems Company, USA). The expression of mRNAs in different groups was calculated and analyzed using the expression of *β*-actin as a reference. All data were calculated using the 2^−*ΔΔ*CT^ method.

### 2.9. Western Blotting (WB)

The uteri were denatured in sample buffer and heated in boiling water for 5 min. Proteins were separated by 10% SDS-PAGE and transferred electrophoretically from the gels to polyvinylidene difluoride (PVDF) transfer membranes. The membranes were incubated for 2 h in a blocking solution containing 5% skim milk and 0.05% Tween-20 in PBS (PBS-Tween). The membranes were washed briefly in PBS-Tween and incubated with c-Met (1 : 1000, CST, #82202), p-c-Met (1 : 1000, CST, #3077), AKT (1 : 1000, Servicebio, GB111114), or p-AKT (1 : 1000, CST, #4060) antibodies at 4°C overnight. The membranes were next washed three times in PBS-Tween using a rotary shaker. The washed membranes were incubated with horseradish peroxidase-conjugated anti-rabbit for 1 h. The membranes were washed again and processed with an enhanced chemiluminescence detection kit (Biosharp, USA) to visualize the proteins recognized by the antibodies, according to the manufacturer's directions.

### 2.10. Statistical Analysis

The statistical software SPSS 23.0 was used for statistical analysis. The quantitative data are expressed as the mean standard deviation of at least three independent experiments. All statistical analyses were performed using Student's two-tail paired *t*-test. *P* < 0.05 is considered statistically significant. The asterisk (∗∗) indicates *P* < 0.01 and the asterisk (∗) indicates *P* < 0.05.

## 3. Results

### 3.1. Characteristics of HGF Gene-Transfected MSCs

**Figure 1:**
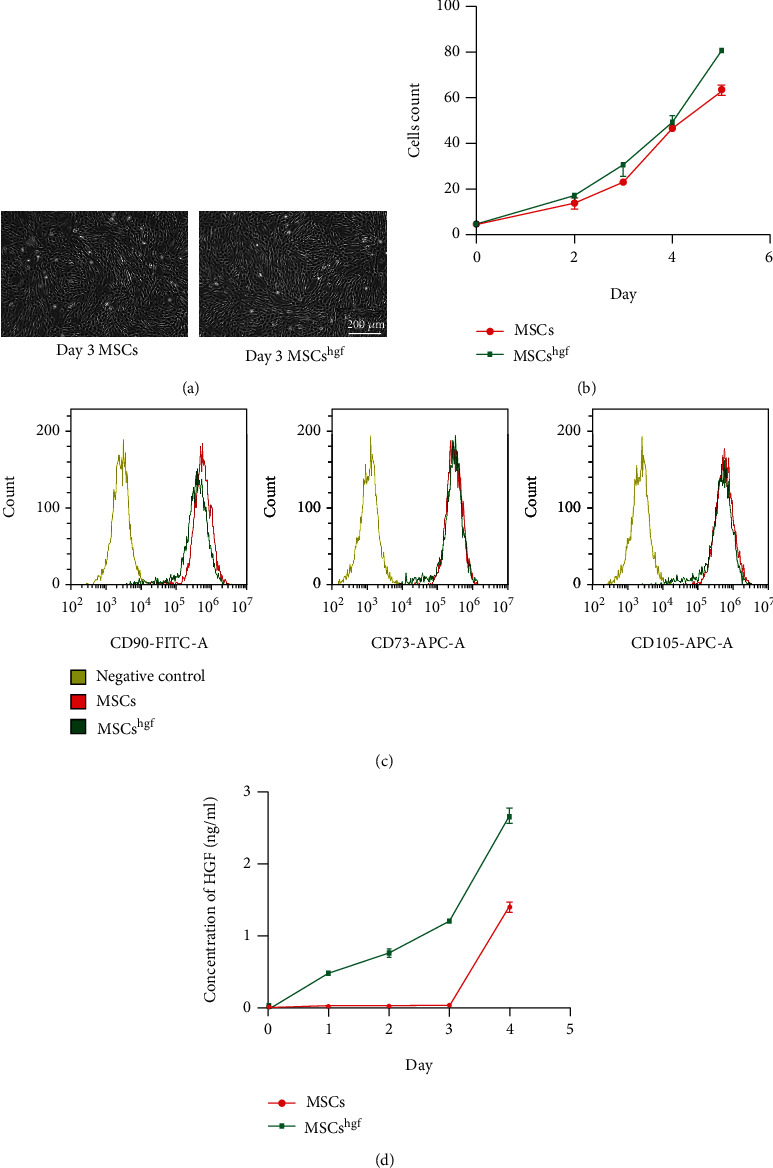
HGF gene-transfected MSCs (MSCs*^hgf^* in the legend) retain the characteristics of original MSCs and significantly increased the expression of the HGF gene in transfected MSCs. (a) Cell morphology of MSCs and HGF gene-transfected MSCs. (b) Cell count of MSCs and HGF gene-transfected MSCs (*n* = 3) at 0, 2, 4, and 6 d. (c) Positive markers (CD73, CD90, and CD105) on the surface of mesenchymal stem cells were tested in MSCs and HGF gene-transfected MSCs. (d) Expression of HGF in MSCs and HGF gene-transfected MSCs (*P* < 0.01, *n* = 3).

To characterize the HGF gene-transfected MSCs, we evaluated cell morphology and cell proliferation. To assess the transfection efficiency, we measured the fluorescence of HGF-GFP-transfected MSCs and the percentage of HGF-GFP-transfected MSCs (Supplementary Figure 1).  Figure 1(a) shows the cell morphology of MSCs and HGF gene-transfected MSCs, with both displaying the same shape as spindle cells. Under the same culture conditions, MSCs and HGF gene-transfected MSCs showed almost the same cell proliferation (Figure 1(b)). To verify the characteristics of mesenchymal stem cells, we checked whether positive markers, including CD73, CD90, and CD105, were all highly expressed in both MSCs and HGF gene-transfected MSCs (Figure 1(c)). Additionally, the negative markers of MSCs, including CD14, CD34, CD45, CD79*α,* and HLA-DR, were negative in both MSCs and HGF gene-transfected MSCs (Supplementary Figure 2). To assess the effects of HGF gene transfection, the concentration of HGF was measured. The concentration of HGF in the cell suspension of HGF gene-transfected MSCs was higher than that of the nontransfected MSCs at 0, 1, 2, 3, and 4 d (Figure 1(d)). These results show that HGF gene-transfected MSCs retained the characteristics of original MSCs and significantly increased the expression of HGF in transfected MSCs.

### 3.2. HGF Gene-Transfected MSCs Recover Normal Morphology in Injured Uteri

**Figure 2:**
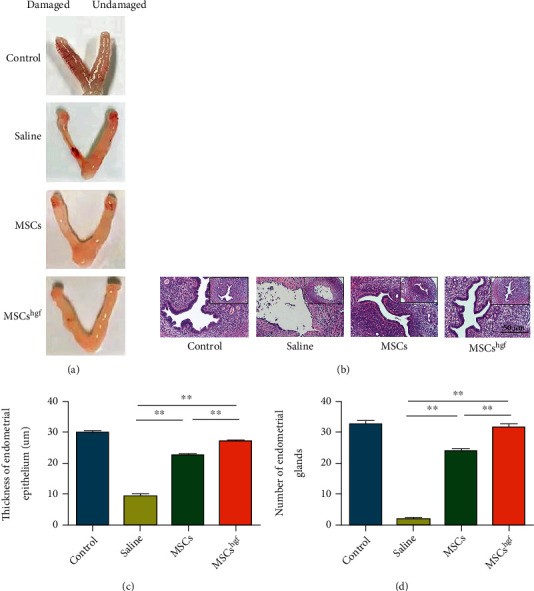
HGF gene-transfected MSCs improve the morphological characteristics and changes of the uterus. (a) Morphological comparison of mouse uterine specimens in each treatment group. (b) H&E staining of mouse uteri in each treatment group (40x). (c) Difference of the thickness of endometrial epithelium in each treatment group (^∗∗^*P* < 0.01, *n* = 10). (d) Difference of the number of glands in each treatment group (^∗∗^*P* < 0.01, *n* = 10).

To verify the effect of HGF gene-transfected MSCs, we established a mouse model of endometrial injury and observed the morphological characteristics of the mouse uterus after HGF gene-transfected MSC therapy. The results show that HGF gene-transfected MSCs improved the appearance and morphological characteristics of the damaged side of the uterus, with the appearance of the uterus similar to the undamaged side (Figure 2(a)). H&E staining showed that the endometrial structure of the HGF gene-transfected MSC group was relatively complete, with the epithelial cells closely arranged and glands clearly visible. In the saline group, the endometrium of the injured side demonstrated a poor morphology, with the thickness of the endometrium clearly thinner, and the epithelium discontinuous and incomplete (Figure 2(b)). Next, the endometrial epithelium thickness was measured, and the number of endometrial glands was counted. Both the epithelium thickness and number of glands in the HGF gene-transfected MSC treatment group were significantly greater than that of the saline group and MSC group (Figures 2(c) and 2(d)).

### 3.3. HGF Gene-Transfected MSCs Improve Proliferation and Decrease Apoptosis of Endometrial Cells

**Figure 3:**
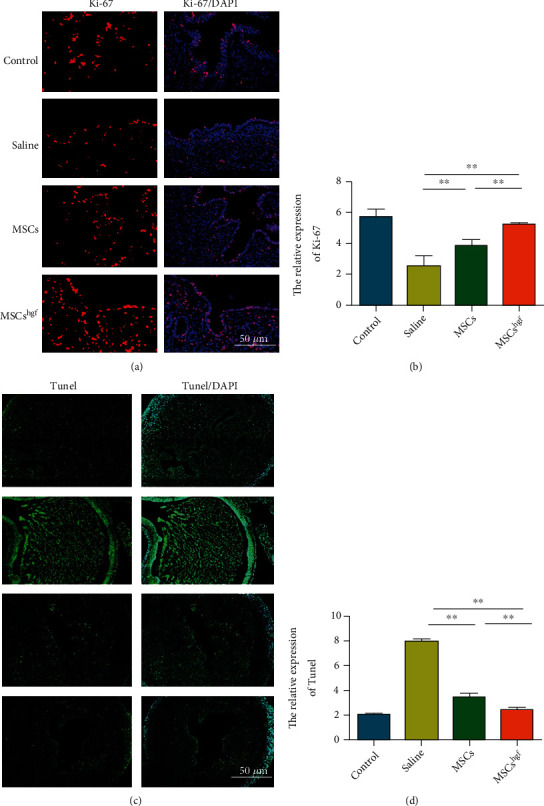
HGF gene-transfected MSCs increase proliferation and decrease apoptosis of endometrial cells in the uterus. (a) Representative images of Ki-67 immunofluorescence in each treatment group. (b) Statistical analysis of Ki-67 immunofluorescence intensity in each treatment group (^∗∗^*P* < 0.01, *n* = 3). (c) Representative images of TUNEL immunofluorescence in each treatment group. (d) Statistical analysis of TUNEL immunofluorescence intensity in each treatment group (^∗∗^*P* < 0.01, *n* = 3).

To verify the repair ability of HGF, we analyzed the proliferation and apoptosis of the epithelial cells. HGF gene-transfected MSCs improved the proliferation ability of damaged endometrial epithelial cells (Figure 3(a)). Through a statistical analysis of Ki-67 fluorescence intensity, we evaluated the cell proliferation effect of HGF gene-transfected MSCs, which was significantly higher than those of that saline and MSC group (Figure 3(b)). A statistical analysis of TUNEL fluorescence intensity quantitatively evaluated the antiapoptotic effect of HGF gene-transfected MSCs (Figure 3(c)). It is significant that the concentration of apoptotic cells in the HGF gene-transfected MSC group was less than both the saline and MSC group (Figure 3(d)).

### 3.4. HGF Gene-Transfected MSCs Improve the Angiogenesis of Damaged Endometrium

**Figure 4:**
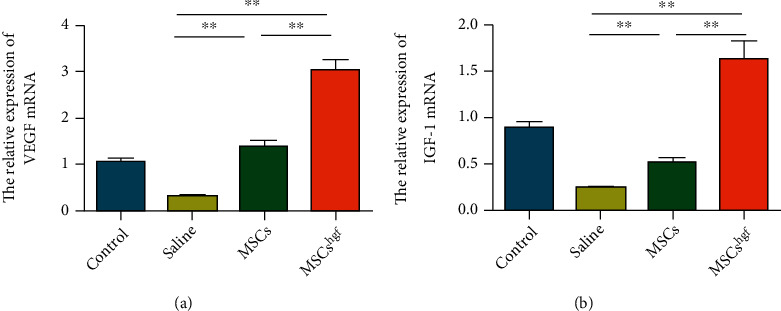
HGF gene-transfected MSCs promote the angiogenesis of damaged endometrium. (a) Relative expression of VEGF mRNA in each treatment group. (b) Relative expression of IGF-1 mRNA in each treatment group (^∗∗^*P* < 0.01, *n* = 3).

To verify the repair effect of HGF on injured endometrial vessels, we detected the expression levels of two angiogenesis-related mRNA: vascular endothelial growth factor (VEGF) and insulin-like growth factor 1 (IGF-1). The mRNA expression of VEGF and IGF-1 in uteri of each group was measured (Figures 4(a) and 4(b)), with the results indicating that HGF gene-transfected MSCs improved the angiogenesis of endometrial epithelial cells. Therefore, HGF gene-transfected MSCs provide an improved vascular repair ability compared to normal MSCs.

### 3.5. HGF Gene-Transfected MSCs Promote the MET Process in Damaged Endometrium

**Figure 5:**
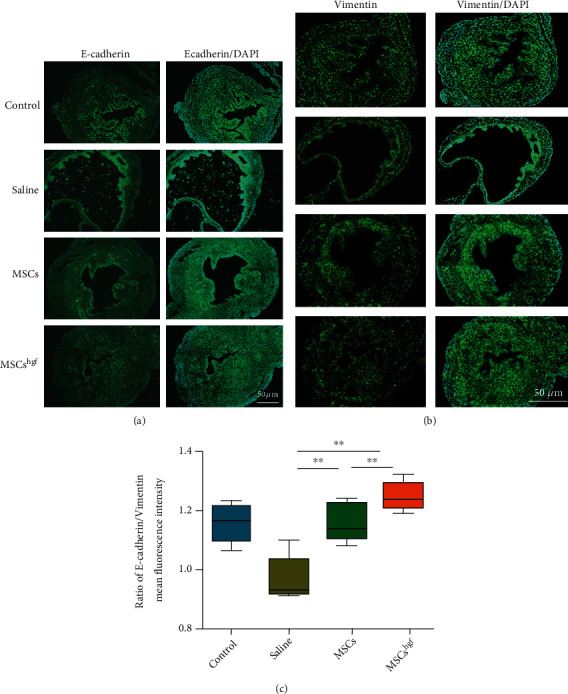
HGF gene-transfected MSCs promote the degree of the MET process in damaged endometrium. (a) Representative images of E-cadherin immunofluorescence in each treatment group. (b) Representative images of vimentin immunofluorescence in each treatment group. (c) The ratio of E-cadherin and vimentin intensity in each treatment group (^∗∗^*P* < 0.01, *n* = 3).

To observe the effect of HGF gene-transfected MSCs on the MET process in damaged endometrium, we used immunofluorescence to label the epithelial marker E-cadherin and mesenchymal marker vimentin (Figures 5(a) and 5(b)). We quantitatively analyzed the ratio of E-cadherin and vimentin fluorescence intensity in each treatment group (Figure 5(c)). The results showed that the process of MET was more active and the degree of epithelial transformation was highest in the HGF gene-transfected MSC group. These results show that HGF plays a significant role in the MET process.

### 3.6. HGF Gene-Transfected MSCs Activate Phosphorylated c-Met and the Downstream Phosphorylated AKT Pathway

**Figure 6:**
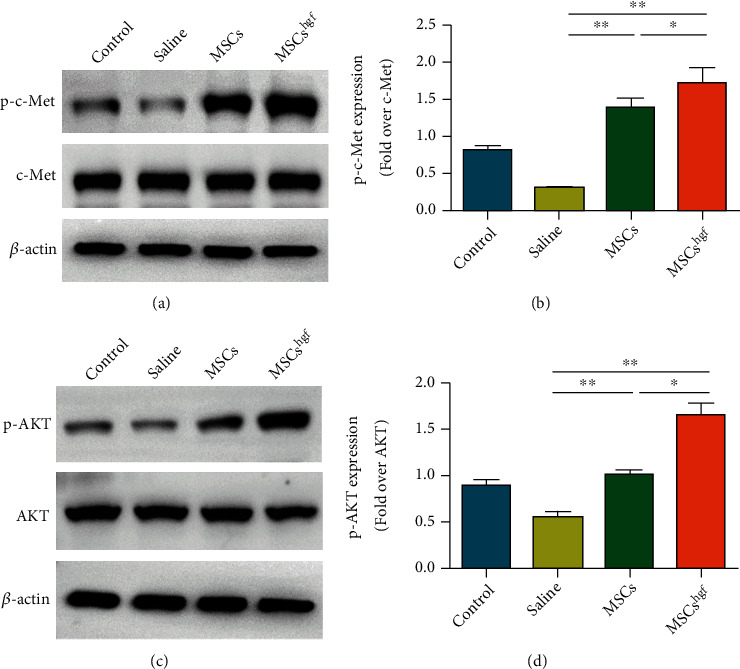
HGF gene-transfected MSCs activate phosphorylated c-Met and the downstream phosphorylated AKT pathway. (a) Representative images of p-c-Met in each treatment group. (b) Densitometric analysis of changes in the abundance of phosphorylated c-Met normalized to c-Met for loading variability. (c) Representative images of p-AKT in each treatment group. (d) Densitometric analysis of changes in the abundance of phosphorylated AKT normalized to AKT for loading variability. (^∗∗^*P* < 0.01, ^∗^*P* < 0.05, *n* = 3).

To explore the mechanism of HGF gene-transfected MSCs on reconstruction and regeneration, we verified the expression of the HGF receptor c-Met and its phosphorylation (p-c-Met) by Western blot and then evaluated the expression of AKT and AKT phosphorylation (p-AKT) in its downstream pathway (Figures 6(a) and 6(b)). The results showed that the expression of p-c-Met in the HGF gene-transfected MSC group was higher than the other groups during the acute inflammation stage following injury, and the expression of p-AKT in its downstream pathway was also higher in the HGF gene-transfected MSCs than the other groups.

## 4. Discussion

In this study, we found that HGF gene-transfected MSCs provided a better therapeutic effect compared to normal MSCs in a mouse model of endometrial damage. Increasing evidence suggests that the therapeutic effects of MSCs mainly depend on their capacity to secrete a variety of growth factors and cytokines [21], including HGF. However, exogenous HGF only has a temporary effect on tissue regeneration, owing to its short half-life in blood circulation [22]. In contrast, MSCs can persistently release HGF, which ensures a continuous expression of HGF in organisms and overcomes the disadvantage of injecting exogenous HGF. In this regard, genetic engineering of MSCs transfected with the HGF gene may improve the intrinsic secretion of HGF, thus improving the endometrial repair potential of these cells in the context of gene therapy.

It is particularly important to generate an ideal HGF gene-transfected MSCs. Even though some viral vectors are confirmed to be safe for clinical use, an improved method that can avoid entirely the integration of viral vectors is required. In this study, we generated HGF-secreting MSCs using minicircle vectors containing only the transgene expression cassette [23–25]. Minicircles are small circular plasmids that carry transgenes. The absence of the bacterial backbone and the nonintegrating transfection system make minicircle vectors an attractive delivery tool in vivo for future clinical applications. Genetically engineering HGF into MSCs via minicircles to treat injured endometrium would be optimal from both a pharmacological and practical perspective [20, 26]. After transfecting with the HGF gene, we analyzed the characteristics and secretion ability of the cells to ensure the safety and effectiveness of the minicircles. By analyzing the cellular activity of MSCs after HGF gene transfection and the characteristic expression of mesenchymal stem cell markers (CD73, CD90, and CD105), it was confirmed that HGF gene transfection did not change the inherent properties of MSCs. Additionally, the ability of MSCs to secrete HGF before and after transfection was also tested. It was found that the ability of MSCs to secrete HGF after transfection was significantly increased. In line with our expectations, HGF gene-transfected MSCs were successfully used for stem cell transplantation. To verify the therapeutic effect of MSCs transfected with the HGF gene on damaged endometrium, we compared the treatment results of ordinary MSCs and HGF gene-transfected MSCs in a mouse model through biochemical, histological, and molecular biological methods. The results show that HGF gene-transfected MSCs have a positive recovery effect on endometrial regeneration and more significantly than ordinary MSCs.

Recent studies have shown that the endometrial tissue of patients with uterine trauma is characterized by vascular closure and microvascular damage. Angiogenesis will appear in damaged endometrial tissue after treatment, suggesting that angiogenesis may improve the regeneration of endometrium [27, 28]. HGF plays an indispensable role in angiogenesis [29, 30]. To explore the mechanism of HGF gene-transfected MSCs in the treatment of endometrial injury, we verified the vascular regeneration ability of the damaged tissue after transplanting with normal MSCs and HGF gene-transfected MSCs. In our experiment, the expression of VEGF and IGF-1 in the endometrium of mice treated with MSCs transfected with the HGF gene was higher than that of mice treated with normal MSCs. This further suggests that HGF gene-transfected MSCs can play a role in angiogenesis by increasing the expression of angiogenesis-related factors.

HGF is also involved in the MET process. It is widely accepted that HGF and its receptor c-Met are involved in the transformation of the epithelium and mesenchyme; however, most of these reports are about tumors or endometriosis [31–34]. The endometrium is the dynamic inner layer of the uterus, composed of a functional layer and basement layer and, histologically, composed of mesenchymal and epithelial cells that undergo monthly proliferation, differentiation, and shedding throughout the menstrual cycle in childbearing age women. In the functional layer of the endometrium, the epithelium and mesenchyme are in a dynamic balance process. When the epithelial tissue of the endometrium is damaged, the mesenchyme will transform to the epithelium to maintain the integrity of the epithelium on the surface of endometrium [35, 36]. Furthermore, MSCs transfected with the HGF gene promote the transformation of mesenchyme to epithelium. The expression of the antigens E-cadherin in epithelial cells and N-cadherin in mesenchymal cells can reflect the degree of MET [37]. Preliminary detection of these two proteins confirmed that HGF transfected-MSCs improved the repair of endometrium through angiogenesis and MET, although the exact mechanism is still unclear. We also analyzed the expression of proteins resulting from HGF binding to its receptor c-Met, activating the phosphorylation of c-Met receptor, and activating the downstream AKT pathway, to verify that MSCs transfected with HGF increased activation of this pathway. This pathway is required for increasing angiogenesis and the MET process [38–40].

To a certain extent, the mouse model of ethanol-induced endometrial injury could represent the endometrial injury, and the ethanol treatment time was demonstrated to be positively correlated with the degree of endometrial damage, fibrosis, inflammation, and vascular trauma. However, the murine reproductive system is very different from that of humans and ethanol-induced uterine damage is clinically rare. The potential mechanism of ethanol-induced endometrial injury cannot completely recapitulate the pathogenesis of clinical IUA, which is the limitation of the animal model [41, 42]. To improve the effect of cell therapy, gene therapy with minicircle gene delivery vectors offers one possible way to meet this demand. The cumulative preclinical results with minicircle gene delivery vectors have yielded promising outcomes in stem cell therapy and stem cell reprograming [43]. Moreover, there are multiple clinical trials using mesenchymal stem cells, and several of them are phase III trials. However, gene therapy with minicircle gene delivery vectors needs more research to ensure its safety before clinical trials can be carried out.

In summary, the present study shows that cell therapy with HGF gene-transfected MSCs significantly promotes endometrial repair at the morphological and molecular levels and that these effects are superior to treatment with normal MSCs. In addition, the enhanced therapeutic effects of HGF gene-transfected MSCs were accompanied by increased cell proliferation and decreased apoptosis. Moreover, MSCs transfected with the HGF gene promoted angiogenesis and the MET process by activating the c-Met pathway following transplantation. These results suggest that cell therapy with HGF gene-transfected MSCs may be a new and effective tool for the treatment of injured endometrium.

## Figures and Tables

**Table 1:** Primer sequences.

Gene name	Primer sequence	
VEGF	Forward 5′-3′	5′-TAGAGTACATCTTCAAGCCGTC-3′
	Reverse 5′-3′	5′-CTTTCTTTGGTCTGCATTCACA-3′
IGF-1	Forward 5′-3′	5′-TATGCTGTTTGAACTTATGCGC-3′
	Reverse 5′-3′	5′-GTTCTCCTCGCTGTAGTAGAAG-3′
*β*-Actin	Forward 5′-3′	5′-GTACGCCAACACAGTGCTGTC-3′
	Reverse 5′-3′	5′-GCTCAGGAGGAGCAATGATCTTG-3′

## Data Availability

The data that support the findings of this study are available upon request from the corresponding author. The data are not publicly available due to privacy concerns or ethical reasons.

## Supplementary Materials

Supplementary Materials Supplementary Figure 1: (A) immunofluorescence image of HGF-GFP-transfected MSCs by fluorescence microscope. (B) Percentage of HGF-GFP-transfected MSCs was detected by flow cytometry. Supplementary Figure 2: (A) HGF gene-transfected MSC negative surface markers CD14, CD34, CD45, CD79*α*, and HLA-DR.
