# Supplementary material for: Therapeutic Effects and Repair Mechanism of HGF Gene-Transfected Mesenchymal Stem Cells on Injured Endometrium
Source: Stem Cells Int. 2022 Apr 5;2022:5744538. doi: 10.1155/2022/5744538 (PMC9005300; doi:10.1155/2022/5744538)
Supplement: Supplementary Materials — Supplementary Figure 1: (A) immunofluorescence image of HGF-GFP-transfected MSCs by fluorescence microscope. (B) Percentage of HGF-GFP-transfected MSCs was detected by flow cytometry. Supplementary Figure 2: (A) HGF gene-transfected MSC negative surface markers CD14, CD34, CD45, CD79α, and HLA-DR. [file 5744538.f1.docx]

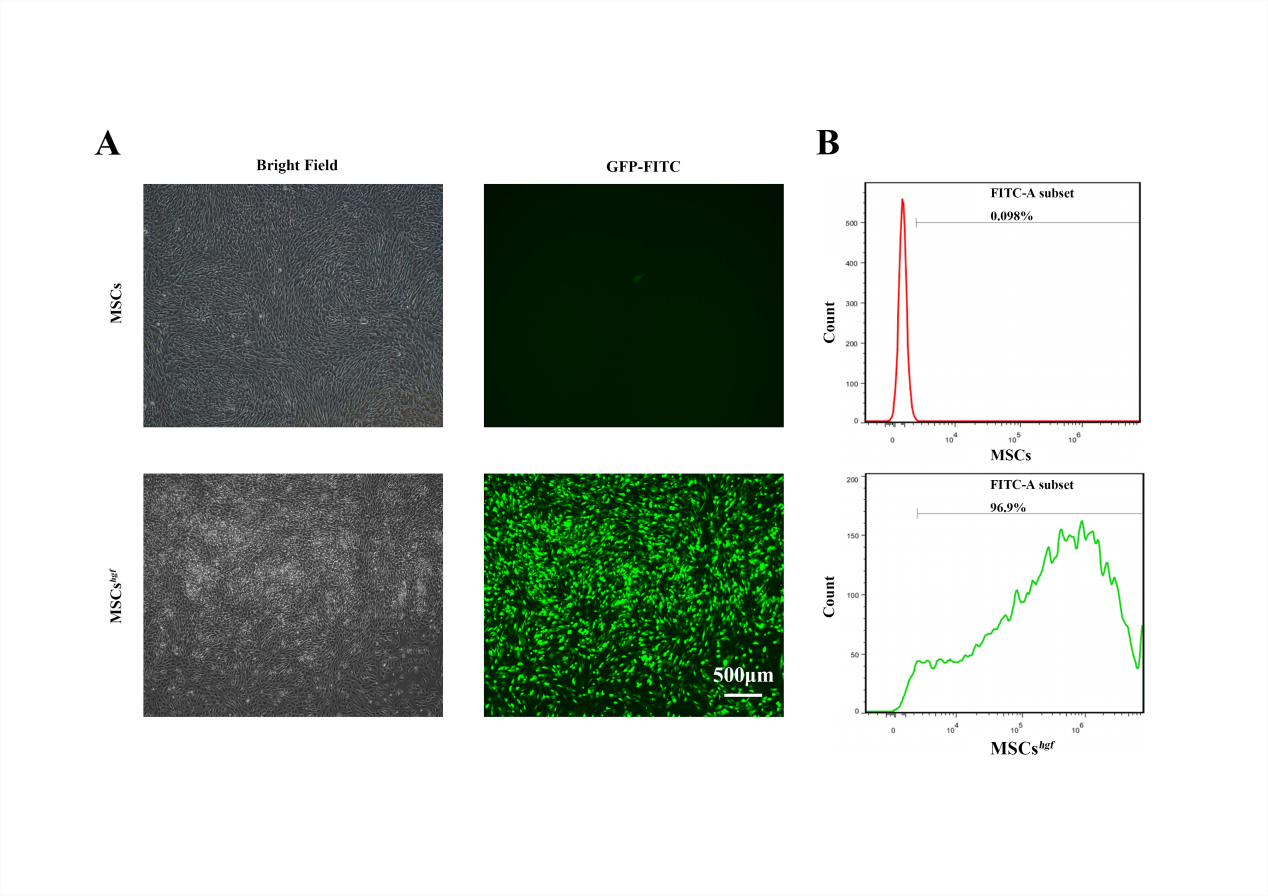


Supplementary figure.1 A. The immunofluorescence image of HGF-GFP transfected MSCs by fluorescence microscope. B. The percentage of HGF-GFP transfected MSCs was detected by flow cytometry.


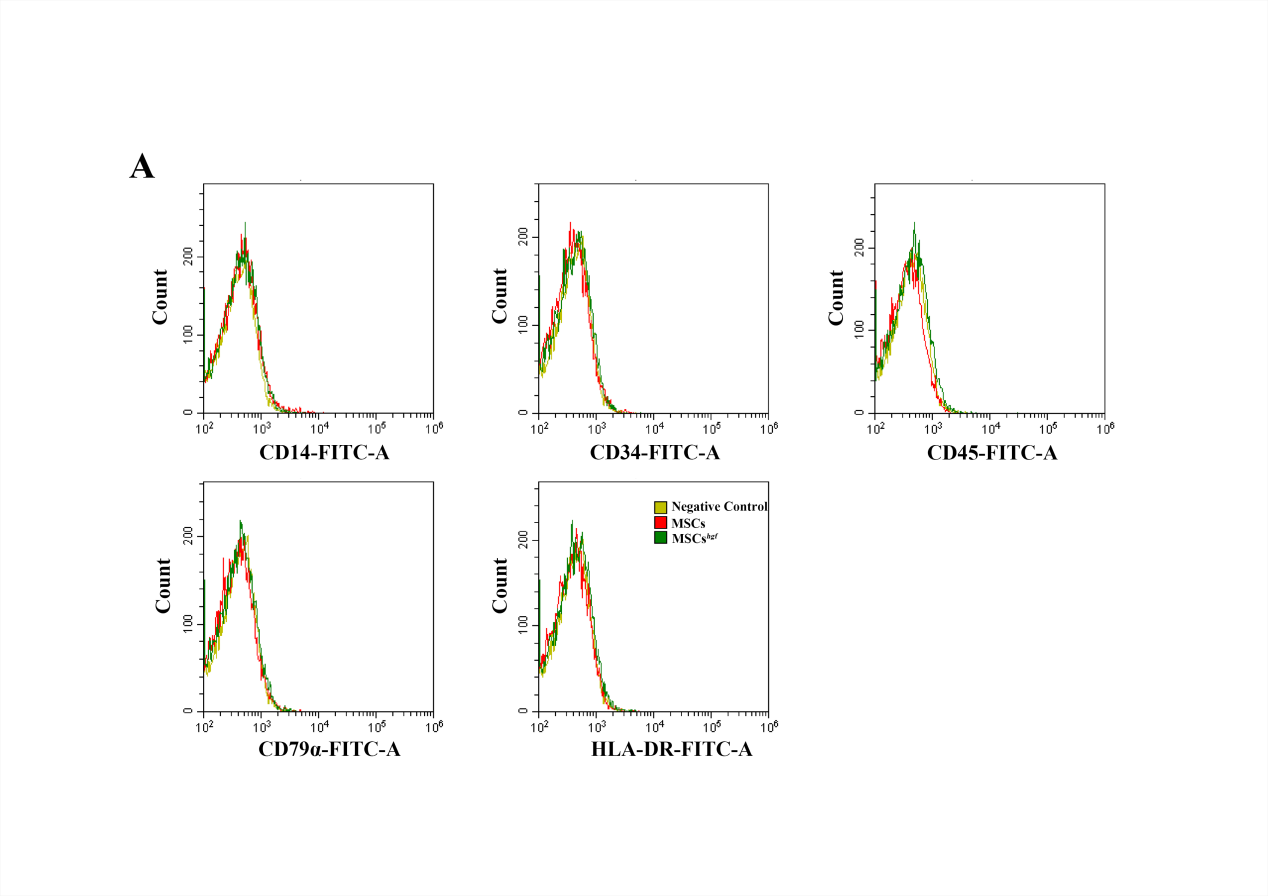


Supplementary figure.2 A. HGF gene-transfected MSCs surface negative marker CD14, CD34, CD45, CD79α and HLA-DR.
